# Biomimetic nanovaccines in cancer therapy: mechanisms, efficacy, and clinical translation

**DOI:** 10.1016/j.mtbio.2025.102116

**Published:** 2025-07-18

**Authors:** Dilinaer Wusiman, Yu Wang, Minghao Wang, Jie Wang, Ruicheng Wu, Zhouting Tuo, Zhipeng Wang, Qingxin Yu, Zhaohong An, William C. Cho, Dengxiong Li, Wuran Wei, Dechao Feng

**Affiliations:** a Department of Urology, The First Affiliated Hospital of Zhejiang Chinese Medical University (Zhejiang Provincial Hospital of Chinese Medicine), Hangzhou, China; b Purdue Institute for Cancer Research, Purdue University, West Lafayette, IN, 47907, USA; c Department of Radiation Oncology, National Cancer Center/National Clinical Research Center for Cancer/Cancer Hospital, Chinese Academy of Medical Sciences and Peking Union Medical College, Beijing, China; d Department of Radiotherapy, The First Hospital of China Medical University, 110167, Shenyang, China; e Department of Urology, Institute of Urology, West China Hospital, Sichuan University, Chengdu, 610041, China; f Department of Urological Surgery, Daping Hospital, Army Medical Center of PLA, Army Medical University, Chongqing, China; g Department of Urology, Sichuan Provincial People's Hospital, University of Electronic Science and Technology of China, Chengdu, China; h Department of Pathology, Ningbo Clinical Pathology Diagnosis Center, Ningbo City, Zhejiang Province, 315211, China; i Department of Pathology, Ningbo Medical Centre Lihuili Hospital, Ningbo City, Zhejiang Province, 315040, China; j Department of Head and Neck Surgery, National Cancer Center/National Clinical Research Center for Cancer/Cancer Hospital, Chinese Academy of Medical Sciences and Peking Union Medical College, Beijing, 100021, China; k Department of Clinical Oncology, Queen Elizabeth Hospital, Kowloon, Hong Kong, China; l Division of Surgery & Interventional Science, University College London, London, W1W 7TS, UK

**Keywords:** Biomimetic nanovaccines, Cancer immunotherapy, Immunogenicity, Nanoscale materials, Clinical implications

## Abstract

Biomimetic nanovaccines have emerged as a promising strategy in cancer therapy, utilizing nanoscale materials that mimic biological systems to elicit robust immune responses. This review delves into the mechanisms by which biomimetic nanovaccines activate the immune system, focusing on their ability to present tumor antigens and stimulate dendritic cells. Various types of biomimetic nanovaccines, including those based on virus-like particles, cell membrane-coated nanoparticles, and peptide-based nanovaccines, are examined. Preclinical studies demonstrating enhanced immunogenicity and anti-tumor effects are highlighted, along with an analysis of current clinical trials assessing the safety and efficacy of these nanovaccines in cancer patients. The review also addresses the technical and regulatory challenges in developing biomimetic nanovaccines and offers insights into future innovations that could facilitate their clinical translation.

## Graphical abstract

**Figure:**
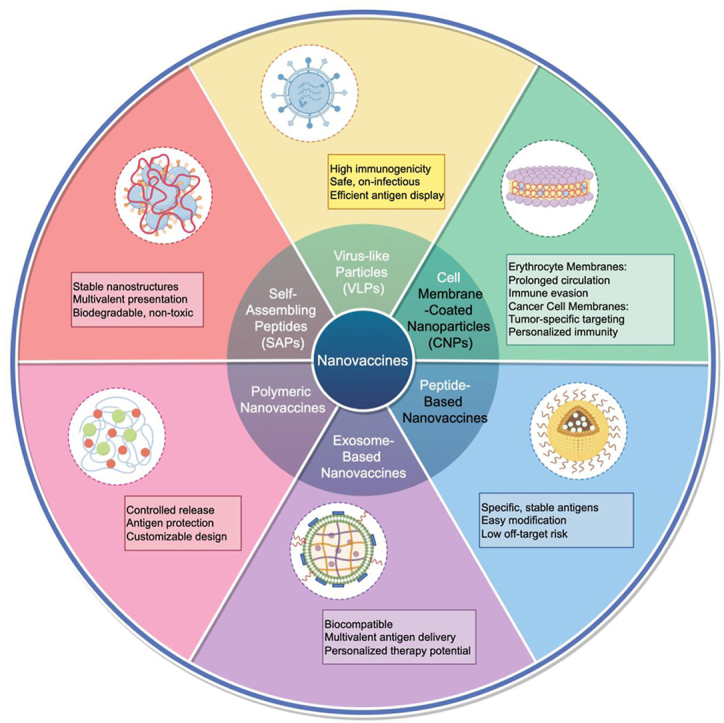

## 1 Introduction

Cancer remains one of the leading causes of death, accounting for almost one in six deaths globally and 22.8 % of deaths from noncommunicable diseases [1,2]. These staggering figures underscore the urgent need for continuous advancements in therapeutic strategies. Traditional cancer therapies, such as chemotherapy, radiation, surgery, and targeted therapy, often face significant limitations, including non-specific targets, considerable side effects, and the potential for tumor recurrence [[3], [4], [5]]. Recently, immunotherapy has emerged as a promising treatment, demonstrating significant efficacy in many cancer patients [[6], [7], [8]]. Researchers are also investigating combination therapies to enhance patient outcomes and prolong survival [9,10]. However, these approaches still encounter challenges, such as immune-related adverse events, treatment resistance, and high costs [11,12]. Consequently, innovative approaches are essential to improve efficacy and patient outcomes, addressing these limitations through more precise, personalized, and less toxic treatment strategies.

**Fig. 1:**
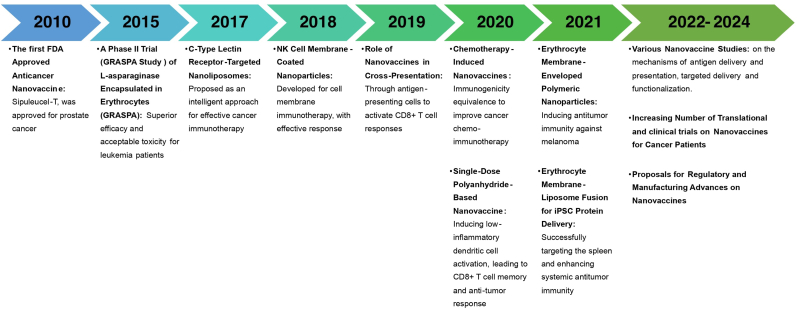
Historical timeline of major nanovaccines development.

Nanovaccines represent a promising frontier in cancer treatment. Traditional nanovaccines leverage nanoscale particles to directly deliver antigens and adjuvants to the immune system, enhancing the body's natural defense against tumors [13,14]. These nanovaccines offer numerous advantages, such as targeted delivery [15], controlled release [16], and the ability to stimulate robust immune responses while minimizing systemic toxicity [17]. They encompass a broad range of nanoparticle platforms, including polymeric nanoparticles, liposomes, inorganic nanoparticles, virus-like particles (VLPs), DNA-based vaccines. Biomimetic nanovaccines, inspired by natural biological systems, are designed to emulate the body's innate mechanisms of pathogen recognition and immune activation [18]. These nanovaccines can be engineered to deliver tumor antigens and immune-stimulating agents in a controlled manner, enhancing the immune system's ability to detect and destroy cancer cells [19]. By incorporating elements such as cell membranes or VLPs, these nanovaccines can improve antigen stability, enhance immune recognition, and facilitate targeted delivery. Recent advancements in nanotechnology and cancer biology have facilitated the development of these sophisticated nanovaccine systems by closely mimicking natural biological processes, addressing the limitations of traditional cancer vaccines, such as limited efficacy and adverse side effects [20]. The progression of nanovaccine development is depicted in Fig. 1.

The fundamental distinction between traditional nanovaccines and biomimetic nanovaccines lies in their foundational design philosophy and material composition, which directly influences their mechanisms of immune activation and clinical performance [21]. Traditional nanovaccines often require additional adjuvants to boost immune responses, as their synthetic nature may not sufficiently stimulate innate immunity [22]. They can also exhibit weak cell-mediated responses, necessitating multiple doses. Biomimetic nanovaccines, however, mimic pathogen structures, enabling them to act as intrinsic adjuvants. For instance, virosomes’ surface antigens interact with host receptors, triggering robust humoral and cellular responses without extra adjuvants [23]. A key advantage of biomimetic nanovaccines is their ability to induce immunogenic cell death (ICD) which releases tumor-associated antigens and further stimulates the immune system [24]. Moreover, integrating adjuvants and other immune-modulating agents into these nanovaccines can amplify the immune response. For instance, agents that generate reactive oxygen species or deplete intracellular antioxidants can enhance the ICD effect, boosting the efficacy of immune checkpoint inhibitors [25]. This approach not only improves response rates but also contributes to improve immune response in recurrent and metastatic tumors, which are significant hurdles in cancer treatment.

This review aims to provide a comprehensive examination of biomimetic nanovaccines in cancer therapy, focusing on their mechanisms, efficacy in preclinical models, and potential for clinical translation. The scope encompasses a detailed analysis of the design and engineering of various biomimetic nanovaccine types, their immunological mechanisms, and their performance in preclinical and clinical settings. The unique contribution of this review lies in its integrative approach, combining insights from recent advancements in nanotechnology, immunology, and clinical research to offer a holistic perspective on the development and application of biomimetic nanovaccines. By highlighting both the opportunities and challenges in this field, this review seeks to guide future research and facilitate the translation of these innovative therapies into clinical practice.

We conducted a structured literature search using databases including PubMed, Web of Science, and Scopus. Keywords such as “biomimetic nanovaccines,” “cancer immunotherapy,” “nanoparticles,” and “clinical translation” were used individually and in combination. Articles published in English from 2015 to 2025 were considered. This review is structured as follows: Section 2 examines the mechanisms of biomimetic nanovaccines, and section 3 covers their design. Section 4 assesses preclinical efficacy, highlighting immunogenicity, anti-tumor effects, and advantages over conventional vaccines. Section 5 discusses clinical trials and translation challenges. Section 6 offers future perspectives and conclusions.

## 2 Mechanisms of biomimetic nanovaccines

This section delves into how biomimetic nanovaccines mimic biological systems to deliver antigens and activate immune responses, laying the foundation for their design and application.

### 2.1 Definition and basic principles

Unlike conventional nanovaccines, which primarily function as synthetic nanocarriers for passive antigen delivery with limited immunomodulatory capacity, biomimetic nanovaccines are engineered to utilize nanomaterials to mimic not only the surface structures of pathogens but also the core functional features of specific pathogens in vivo, including their immune recognition motifs, invasion mechanisms, and multi-signal stimulation patterns [26]. These precisely engineered nanoparticles not only mimic the surface and antigenic features of pathogens but also replicate their ability to activate pattern recognition receptors (PRRs), allowing targeted interaction with antigen-presenting cells like dendritic cells (DCs), macrophages, and B cells. This structural and functional mimicry allows biomimetic nanovaccines to effectively trigger immune responses by imitating how pathogens are naturally captured and processed, boosting antigen uptake by antigen presenting cells (APCs) [27]. Meanwhile, their co-delivery of tumor antigens (including tumor-associated antigens, neoantigens, or antigen-encoding mRNA) and immunostimulants like toll like receptor (TLR) agonists recapitulates the multi-component stimulation of pathogens [28].

Conventional nanovaccines typically rely on synthetic carriers for antigen encapsulation, featuring relatively passive targeting and single-mode immune activation [29,30]. In contrast, the most pivotal advantage of biomimetic nanovaccines lies in their ability to recapitulate the multi-dimensional immune stimulation patterns of natural pathogens, thereby overcoming the inherent limitations of traditional vaccines in activating coordinated, potent, and sustained immune responses. Traditional vaccines, including inactivated pathogens, subunit vaccines, and conventional nanovaccines, typically rely on a single antigen or a basic antigen-adjuvant mix, leading to limited immune activation [31]. They often prioritize humoral immunity (via antibody production) or, in the case of certain nanovaccines, limited cellular immunity, with inefficient targeting of APCs and suboptimal activation of intracellular signaling pathways.

### 2.2 Antigen delivery and presentation

**Fig. 2:**
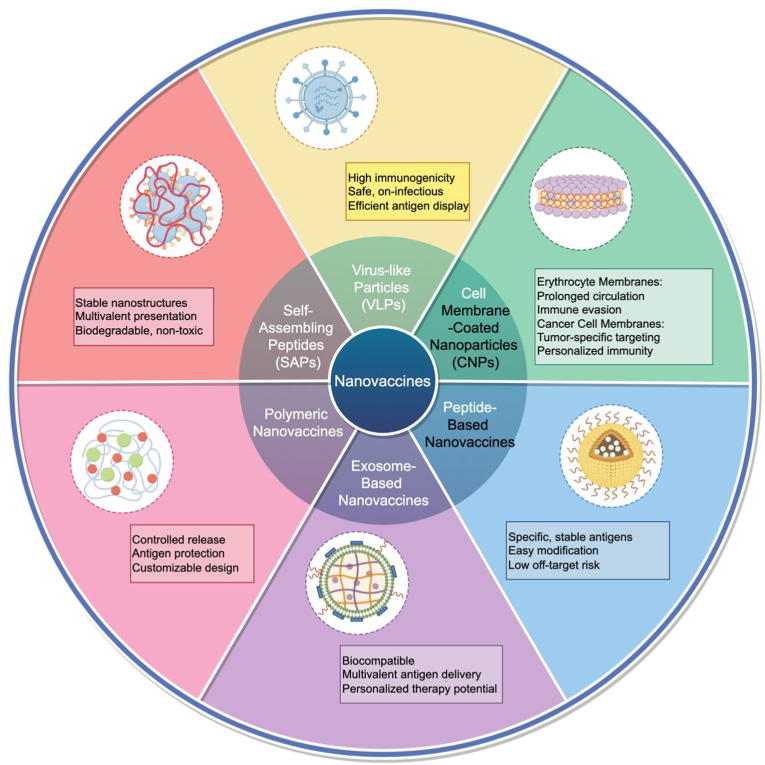
**Mechanism of antigen presentation and immune activation by nanovaccines.** Immature dendritic cells (DCs) internalize nanovaccines, leading to maturation and antigen presentation. MHC I activation primes CD8^+^ T cells, resulting in cytotoxic T cell-mediated tumor cell killing. Concurrently, MHC II activation stimulates CD4^+^ T cells, which assist in B cell activation and antibody production.

Biomimetic nanovaccines achieve their potent anti-tumor efficacy through a tightly integrated system that couples the specificity of tumor antigens with a pathogen-mimetic machinery for immune activation, ensuring each step-in antigen delivery, processing, and presentation reinforces the next (Fig. 2). Central to this system is the co-localization and co-delivery of diverse tumor antigens, including tumor-associated antigens, mutation-derived neoantigens, and mRNA-encoded antigens, with immunostimulants, a design that leverages the unique properties of each antigen to amplify cross-presentation, the linchpin of CD8^+^ T cell-mediated cytotoxicity [32]. Neoantigens, as cancer-specific peptides absent in normal cells, rely on precise intracellular trafficking to evade immune tolerance and trigger specific responses. Their delivery exemplifies the nanovaccine's mechanistic precision: platforms like sHDL-Ag/CpG nanovaccines encapsulate neoantigen peptides with CpG adjuvants, targeting DCs via receptor-mediated endocytosis [33]. After internalization, the acidic endosome causes the sHDL scaffold to change shape, while CpG binds TLR9, triggering MyD88 recruitment and downstream signaling. This signaling cascade upregulates V-ATPase and NOX2, destabilizing endosomal membranes to facilitate neoantigen escape into the cytosol [34,35]. E3 ligases tag the peptides for ubiquitination, targeting them to the immunoproteasome, which upregulated by IFN-γ, to generate 8–10 amino acid fragments [36]. The fragments are transported into the endoplasmic reticulum (ER) by the TAP1/TAP2 complex, where they bind to major histocompatibility complex (MHC) class I molecules with help from chaperones like tapasin and calreticulin, which ensure proper folding and strong peptide binding [37,38]. The MHC I-neoantigen complexes move through the Golgi to the DC surface, where they activate CD8^+^ T cells by binding their TCRs, with help from CD80/CD86 co-stimulation and IL-12, leading to cytotoxic T cell activation [39]. These CTLs then recognize neoantigen-MHC I complexes on tumor cells, inducing apoptosis via perforin-mediated membrane pores and granzyme B-triggered caspase activation [40,41].

In contrast, mRNA vaccines capitalize on their ability to encode full-length antigens, streamlining antigen production by bypassing pre-processing steps. Encapsulating mRNA in biocompatible carriers like lipid nanoparticles or nanoerythrosomes protects it from RNases and directs it to DCs. Nanoerythrosomes, made by fusing tumor antigens with red blood cell membranes, use CD47 to avoid macrophages and bind DC receptors like DEC-205 for uptake [42]. Once internalized, the carrier's pH-sensitive lipids protonate in acidic endosomes, reducing membrane charge repulsion to fuse with endosomal membranes, releasing mRNA directly into the cytosol [38]. Cytosolic ribosomes translate mRNA into antigenic proteins, which either undergo proteasomal degradation (mirroring the neoantigen pathway) to generate MHC I-binding peptides or are secreted for re-internalization, amplifying cross-presentation. This direct cytosolic access speeds up antigen production compared to DNA vaccines, which need nuclear entry, leading to quicker PLC engagement and stronger CD8^+^ T cell activation [43].

## 3 Design and engineering of biomimetic nanovaccines

Building on the mechanisms by which biomimetic nanovaccines activate the immune system, their design and engineering are critical for optimizing antigen delivery and immune response. This section explores the diverse types of biomimetic nanovaccines and their surface functionalization strategies, highlighting how their structural and functional mimicry of biological systems enhances therapeutic efficacy.

### 3.1 Types of biomimetic nanovaccines

**Fig. 3:**
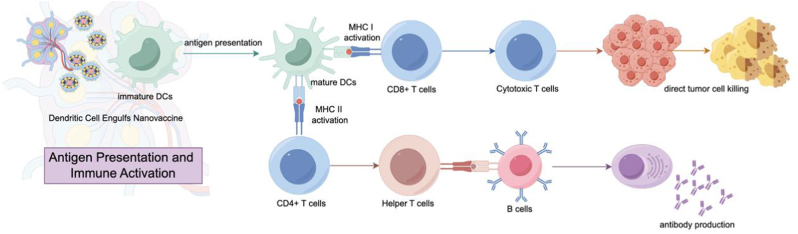
**Types and advantages of nanovaccines.** The primary types of biomimetic nanovaccines include virus-like particles (VLPs), cell membrane-coated nanoparticles (CNPs), peptide-based nanovaccines, exosome-based nanovaccines, polymeric nanovaccines, and self-assembling peptides (SAPs) nanovaccines.

Biomimetic nanovaccines are an innovative class of vaccines that include various types designed to mimic natural biological processes and structures. These nanovaccines leverage the advantages of nanoscale materials to enhance immune responses and provide improved protection against diseases. The primary types of biomimetic nanovaccines include VLPs, cell membrane-coated nanoparticles (CNPs), peptide-based nanovaccines, exosome-based nanovaccines, polymeric nanovaccines, and self-assembling peptides (SAPs) nanovaccines (Fig. 3).

VLPs are nanoscale structures assembled from viral capsid proteins, retaining the surface antigen characteristics of viruses but lacking the viral genome. They effectively mimic the appearance and surface features of natural virus particles [44]. VLPs can tightly and efficiently bind to B cell receptors and be recognized by natural IgM antibodies, activating the classical complement pathway. Simultaneously, the internal antigens of VLPs can be presented to T cells, eliciting a cellular immune response [45,46]. This dual immune activation mechanism enhances nanovaccine efficacy. VLPs can activate memory immune cells, enabling a rapid and robust immune response upon re-exposure to the pathogen. For example, a liver cancer nanovaccine constructed using HBV capsid proteins with inserted liver cancer antigens activates the immune system to recognize and attack liver cancer cells, inhibiting tumor growth and metastasis [47].

CNPs involve extracting complete cell membranes and coating them on nanoparticle surfaces. This approach utilizes membrane proteins to prevent antigen degradation in vivo, extending antigen stability [33]. CNPs can mimic the function of natural APCs like DCs and artificial APCs (aAPCs), promoting cross-presentation via the MHC-I pathway and enhancing immune responses [48]. Additionally, membrane-coated nanoparticles can control drug release by altering membrane permeability or utilizing channel proteins. For instance, leukocyte membrane-coated nanoparticles can evade host immune detection, enabling prolonged circulation and reduced clearance, enhancing immune evasion and promoting tissue repair [49].

Nanoparticles can exploit the inherent biocompatibility and prolonged circulation time of erythrocyte membranes, allowing for extended antigen presence and improved targeting of APCs. Once internalized by APCs, these nanoparticles promote antigen cross-presentation, leading to a strong cytotoxic T-cell response. One study demonstrated that erythrocyte membrane-coated polymeric nanoparticles carrying melanoma-associated peptides significantly enhanced tumor growth inhibition, prolonged survival in melanoma models, and increased CD8^+^ T-cell infiltration in the tumor [50,51]. Zhai et al. employed erythrocyte membranes fused with liposomes to deliver induced pluripotent stem cell proteins, successfully targeting the spleen and enhancing systemic antitumor immunity [52]. These findings indicate that erythrocyte membrane-coated nanovaccines, by combining prolonged antigen delivery with potent immune stimulation, play a crucial role in improving cancer immunotherapy.

Cancer cell membrane-coated nanoparticles (CCNPs) leverage the unique properties of cancer cell membranes to deliver tumor antigens and overcome immune evasion. These nanoparticles trigger both innate and adaptive immune responses, particularly through cytotoxic T lymphocytes and natural killer (NK) cells, ultimately leading to tumor destruction. Yang et al. [53] utilized mannose-modified cancer cell membranes to encapsulate nanoparticles loaded with immunoadjuvants such as imiquimod, resulting in a robust tumor-specific immune response. When combined with immune checkpoint inhibitors like anti-programmed cell death protein 1 (PD-1) antibodies, the synergistic effect between the nanovaccine and checkpoint inhibitors reverses the immunosuppressive tumor microenvironment (TME) and promotes long-term immune memory to prevent cancer recurrence, thereby improving overall therapeutic efficacy. An innovative example is the “Trojan horse” nanoparticle-delivered cancer cell membrane vaccine, which employs a layered double hydroxide nanoparticle platform. This strategy masks immune evasion proteins on the cancer cell membrane using bovine serum albumin and targets APCs through mannose modification, effectively overcoming immune escape [54]. This approach has been shown to enhance immune responses and inhibit tumor growth in vivo.

Peptide-based nanovaccines consist of peptide antigens, nanoparticle carriers, and adjuvants. Peptide antigens, short chains of amino acids, are the primary active components of nanovaccines [55]. Nanoparticle carriers enhance the stability and bioactivity of peptide antigens and deliver them to immune cells. Common carriers include nanomicelles, nanofibers, and carbon nanotubes. Adjuvants, such as berberine and CpG adjuvants, enhance immune responses by promoting antigen uptake and internalization and DC maturation, improving cross-presentation [56,57]. Nanoparticles offer a unique and efficient delivery platform, enhancing the immunogenicity and stability of peptide antigens. Depending on their size, DCs can internalize nanoparticles or transport them to lymph nodes for immune activation. For instance, fusion peptides combining antimicrobial peptides and tumor antigen epitopes can form nanoparticles (8FNs) that, when administered in vivo, elicit strong tumor antigen-specific CD8^+^ T cell responses, effectively inhibiting tumor growth [58].

Exosome-based nanovaccines represent a promising and innovative approach in cancer immunotherapy, particularly in the development of biomimetic nanovaccines for cancer treatment. As cell-derived vesicles, exosomes possess unique capabilities in regulating immune responses due to their ability to present antigens and stimulate both innate and adaptive immunity [59]. In the context of cancer, exosome-based nanovaccines show great potential in overcoming the immunosuppressive TME by enhancing antigen presentation. For instance, macrophage tumor chimeric exosomes have been demonstrated to efficiently accumulate in lymph nodes and tumors, where they induce T-cell activation and modulate the TME, ultimately reducing tumor growth in preclinical models of lymphoma and breast cancer [60]. Similarly, research by Xu et al. demonstrated that neoantigen-loaded exosomes, when combined with anti-PD-1 therapy, led to complete tumor remission in mice by enhancing DCs uptake and expanding T-cell responses [61]. Therefore, macrophage tumor chimeric exosomes exhibited the ability to accumulate in lymph nodes and tumors, activate immune responses, and improve the TME. These findings underscore the multifaceted role of exosome-based nanovaccines in generating robust immune responses, with their biocompatibility and ability to target specific cells potentially leading to more effective cancer immunotherapies.

Polymeric nanoparticles represent a bioinspired approach to vaccine development [62]. These polymeric structures, self-assembled from amphiphilic polymers, exhibit significant advantages in delivering antigens and adjuvants to DCs, thus initiating robust immune responses [63]. For instance, glycol-targeted cationic lipid-polymer hybrids have been shown to co-deliver antigens and TLR agonists, such as imiquimod and monophosphoryl lipid A, improving antigen uptake and cross-presentation, ultimately enhancing CD4^+^ and CD8^+^ T-cell activation [64]. Moreover, these nanovaccines encapsulate antigens and dual TLR agonists, synergistically stimulating the immune system. Chimeric cross-linked polymers encapsulating doxorubicin and photosensitizers, when used in conjunction with laser irradiation, have been shown to induce ICD [65]. This process not only enhances the presentation of TAAs but also stimulates effective antitumor immune responses, particularly in colorectal cancer models. Similarly, pH-sensitive polymers loaded with gold nanoparticles and doxorubicin have demonstrated responsiveness to acidic tumor environments, enhancing the immunogenicity of dying tumor cells and promoting effective antitumor immune responses, as shown in glioblastoma treatments [66]. These innovations highlight the potential of polymeric nanovaccines to induce ICD and serve as in situ DC vaccines, transforming tumors into personalized vaccines that enhance T-cell-mediated immunity and revolutionize cancer treatment.

SAPs provide a framework for forming nanoscale structures with tumor antigens and immunostimulatory molecules. These SAP-based nanomaterials exhibit high stability in physiological environments, ensuring sustained antigen release and prolonged immune activation [67]. SAP nanovaccines present antigens in a multivalent manner, triggering both humoral and cellular immune responses, which leads to effective antitumor activity and immunological memory. For instance, by combining a novel self-adjuvant containing the tumor-associated antigen MUC1 with MUC1 and helper T-cell epitopes, the nanovaccine induced a robust antigen-specific IgG response in mice, capable of recognizing MUC1 on cancer cells and inhibiting tumor progression [68]. Furthermore, Baharom and colleagues encapsulated the Reps1 antigen within a self-assembling nanoparticle vaccine (SNP-7/8a). The SNP-7/8a platform, which incorporates peptide-TLR-7/8a conjugates, is designed for the efficient delivery of peptide antigens and Toll-like receptor agonists, specifically targeting DCs for antigen presentation [69]. The structural stability and self-assembling properties of this vaccine further enhance its immunogenicity, demonstrating potential as a next-generation cancer vaccine.

### 3.2 Surface functionalization

#### 3.2.1 Targeted delivery using ligands and antibodies

Surface functionalization of nanovaccines typically relies on the modifiability of nanoparticle carriers. Using ligands for targeted delivery is a common method, as ligands can bind to specific receptors, effectively targeting immune cell types and optimizing delivery efficiency [70]. This approach enhances nanovaccine specificity and reduces side effects. Wamhoff et al. utilized Langerin ligands to selectively deliver nanoparticles to Langerhans cells in vitro. Antibodies, with their high specificity, achieve targeted delivery to pathogens or tumor cells [71]. Advanced delivery carriers like lipid nanoparticles can be functionalized with ligands for targeted delivery to specific cell receptors. This method allows lipid nanoparticles to efficiently deliver mRNA nanovaccines to APCs, significantly enhancing antigen stability and immunogenicity [72]. Unlike conventional mRNA vaccines, self-amplifying RNA vaccines contain a 9-kb self-amplifying RNA encapsulated within lipid nanoparticles, which substantially increases immunogenicity compared to unformulated RNA [73]. Clinically, LNP-mRNA vaccines have shown great promise. For example, the 113-O12B LNP system has shown a special ability to target lymph nodes, inducing a strong response from CD8^+^ T cells to melanoma in preclinical models. This suggests a reduced risk of tumor exotoxicity, a common concern for systemic immunotherapy [74].

#### 3.2.2 Cell membrane coating

Coating nanoparticles with membranes from tumor or immune cells mimics the source cell's surface features, including TAAs, glycoproteins, and lipids, to boost targeting, immune evasion, and immunogenicity [53]. This strategy depends on maintaining membrane integrity, careful characterization, and engaging PRRs. These preserved surface features enable specific interactions with PRRs on immune cells, driving both targeting and immune activation. Tumor cell membrane coatings, rich in TAAs and damage-associated molecular patterns, are recognized by TLR2/4 on DCs via lipidated antigens or glycans, triggering MyD88-dependent signaling to promote DC maturation, cytokine secretion, and enhanced antigen uptake. For example, nanovesicles from senescent cancer cell membranes, which retain TNF-α and TAAs, deliver these molecules to DCs. TAAs activate TLR2/4 to trigger inflammation, while cytokines boost STING-driven type I interferon production, together priming tumor-specific T cells [75,76]. Concurrently, immunosuppressive molecules on tumor membranes can be masked during coating, reducing their ability to inhibit T cell activation and allowing unimpeded PRR-mediated stimulation [77]. Immune cell membrane coatings, such as those from macrophages, leverage proteins like CD47, which binds signal regulatory protein alpha on phagocytes to downregulate pro-phagocytic PI3K/Akt signaling, mimicking “self-recognition” to evade macrophage clearance and prolong circulation [78,79]. This immune evasion ensures prolonged exposure of nanoparticles to target cells, where glycans on immune cell membranes engage C-type lectin receptors on DCs, facilitating targeted uptake and antigen presentation via MHC class I/II pathways.

## 4 Efficacy of biomimetic nanovaccines in preclinical studies

With an understanding of the design principles behind biomimetic nanovaccines, evaluating their performance in preclinical studies provides insight into their therapeutic potential. This section examines the immunogenicity, anti-tumor effects, and comparative advantages of these nanovaccines in various tumor models.

### 4.1 Enhanced immunogenicity and lower toxicity

The advent of biomimetic nanovaccines has revolutionized the field of immunotherapy, offering a promising approach to enhance efficacy and safety [56]. In recent years, increasing evidence, particularly in vitro studies, have demonstrated the effectiveness of biomimetic nanovaccines, highlighting their potential to induce robust and specific immunogenicity [80]. In vitro and in vivo studies are critical in the early stage of biomimetic nanovaccine development, allowing researchers to evaluate the immunogenicity of new nanovaccine candidates in a controlled environment. In preclinical studies, biomimetic nanovaccines, leveraging the principles of biomimetics, have shown remarkable potential in these studies [81]. The following sections will delve into the key findings and methodologies employed in these studies.

The design of biomimetic nanovaccines involves mimicking biological structures or processes to enhance their interaction with the immune system. Techniques such as lipid cross-linking, polymer-lipid conjugation, self-assembly, layer-by-layer deposition, and nanoparticle engineering have been employed to create nanovaccines that can effectively deliver antigens and adjuvants to immune cells, allowing for enhanced stability and biological efficacy [[82], [83], [84]]. These fabrication processes ensure that the nanovaccines are biocompatible, stable, and capable of eliciting a targeted immune response [85].

One of the key advantages of biomimetic nanovaccines is their ability to enhance antigen presentation. Pre-clinical studies have shown that these nanovaccines can effectively deliver antigens to APCs, such as DCs, leading to a more potent immune response [[86], [87], [88]]. In addition, the incorporation of adjuvants within the nanovaccine structure can further boost the immunogenicity, providing a synergistic effect that enhances the overall efficacy of the nanovaccine [83].

In vitro and in vivo studies have demonstrated that biomimetic nanovaccines can interact with various immune cells, including macrophages, DCs, NK cells, T cells, and B cells to exert the immunogenicity [86,87,89]. These interactions are crucial for initiating and modulating immune responses. Xia et al. have reported on a pH-/enzyme-responsive TLR7/8 agonist-conjugated nanovaccine (TNV) that intelligently responds to the acidic environment and cathepsin B in the endosome [90]. TNV has been observed to accurately release TLR7/8 agonists and activate its receptor signaling at the endosomal membrane, thereby stimulating DC maturation and provoking specific cellular immunity. In a preclinical melanoma mouse model, TNV administration prevented tumor formation in 80 % of cases. In established tumors, TNV suppressed growth by around 70 % compared to controls. TNV reduced tumor volume by around 60 % and extended survival by >50 % versus free agonist or placebo in colon cancer mice model. The endosome-targeted responsive nanoparticle strategy provides a potential delivery toolbox of adjuvants to advance the development of anti-tumor nanovaccines.

Given that safety is also a paramount concern in nanovaccine development, in vitro studies have been instrumental in assessing the cytotoxicity and immunotoxicity of biomimetic nanovaccines [91]. Recent preclinical studies have shown that, compared to traditional vaccines, nanovaccines generally exhibit lower toxicity and are well-tolerated by immune cells, making them a safer alternative [92,93].

Taken together, in vitro and in vivo studies have provided valuable insights into the potent immunogenicity, anti-tumor effectiveness, and favorable safety of biomimetic nanovaccines. The data gathered from these studies are instrumental in guiding the design and optimization of these nanovaccines for clinical trials in the future. As the field continues to evolve, further research is needed to fully understand the mechanisms underlying the enhanced immunogenicity of biomimetic nanovaccines and to translate these findings into effective clinical therapies.

### 4.2 Effective anti-tumor immunity and survival benefits

Nanovaccines utilized biomimetic principles to enhance the interaction with the immune system, aiming to provide more effective and targeted treatments, and have shown significant promise in recent preclinical research. Matos et al. [94] proposed a nanovaccine strategy to activate the host immunity by delivering peptide antigens, adjuvants, and transforming growth factor (TGF)-β regulators using a polyoxazoline (POx)-poly(lactic-co-glycolic) acid (PLGA) nanoparticle. This approach also modulates the tumor-associated macrophages (TAM) function and blocks the anti-PD-1. More importantly, POx-Mannose nanovaccines elicit stronger antigen-specific T-cell responses, resulting in a more effective control of tumor growth via a CD8^+^ T cell-dependent mechanism [94]. Combining the POx-Mannose nanovaccine with pexidartinib, which is a modulator of the TAM function, effectively restricts MC38 tumor growth and synergizes with PD-1 blockade to control MC38 and CT26 tumor growth and improve survival, as further validated in the B16F10 melanoma model. In this preclinical model, the combination inhibits both TAM- and PD-1-induced immunosuppression and holds promising potential for improving immunotherapy outcomes in patients with solid cancer.

In addition, most researchers chose the TLR agonists, including CpG oligonucleotides, MPLA, R837, and R848, as immunoadjuvants to stimulate anti-tumor immune responses in preclinical studies. Liu et al. [95] proposed an in situ nanovaccine strategy based on a cationic peptide with cholesterol-modified, DP7-C, which is desirable for anti-tumor immunotherapy because of its convenience and capacity to target tumor antigens. This nanovaccine includes cocktail small interfering RNAs and immunologic adjuvant CpG ODNs, demonstrating a synergistic effect in inducing tumor cell death, promoting antigen presentation, and relieving immune suppression within the TME [95]. In the both CT26 (hot) and B16F10 (cold) tumor model mice, this nanovaccine could target primary tumors, enhance immune responses to anti-PD-1 treatments by converting cold tumors into hot ones, and modulate systemic immunity to inhibit metastasis [[95], [96], [97]]. This preclinically tested nanovaccine generated specific tumor cell antigens that stimulated robust anti-tumor immune activities, providing an alternative approach for developing novel cancer therapy [84,98].

Currently, anti-tumor immunity elicited by biomimetic nanovaccines has been extensively studied, with a focus on cytokine profiles and T-cell responses. Zhang et al. indicated T-cell immunoglobulin mucin (TIM)-3 blockade could alleviate T cell exhaustion and trigger DCs inflammasome activation, playing crucial role in anti-tumor immunity [99]. They developed a biomimetic nanovaccine edited with long noncoding RNA (lncRNA) and combined with anti-TIM-3 to facilitate dual-effect antigen cross-presentation and mitigate immunosuppression for enhanced pharmacokinetic profile and immune cell infiltration in the TME. LncRNA editing could induce major histocompatibility complex I and tumor immunogenicity in the tumor cell membrane to encapsulate anti-TIM-3, thereby forming LCCT nanoparticles, which were embedded in an alginate-based hydrogel to suppress postsurgical tumor relapse. LCCT retained the efficacy of anti-TIM-3 blockade in both DCs and CD8^+^ T cells (exceeding 75 %). The integrated anti-TIM-3 augmented endocytosis of LCCT in DCs, amplifying inflammasome activation and antigen cross-presentation, which further stimulated effector and memory-precursor CD8^+^ T cells against tumors and thereby improved the therapeutic benefits [100].

In vitro and in vivo studies also have shown that these nanovaccines can induce Th1 and Th17 cellular immune, characterized by the production of various cytokines such as IFN-γ and TNF-α [101,102]. Qiao et al. further constructed a manganese and aluminum dual-adjuvant antigen co-delivery system (MnO2-Al-OVA) nanoparticles to induce effective Th1 type immune responses by activating cyclic guanosine phospho-adenosine synthase (cGAS)-interferon gene stimulator protein (STING)-IFN-I pathway [103]. This type of response, observed in preclinical models, is crucial for activating cytotoxic T cells, which are essential for targeting and killing cancer cells [89,104]. Additionally, the presence of memory T cells has been observed, suggesting long-lasting immunity post-vaccination.

### 4.3 Comparative analysis with conventional vaccines

The preclinical studies of biomimetic nanovaccines have yielded promising results, demonstrating their potential in decreasing toxicity, modulating immunity and improving therapeutic efficacy, and therefore providing a more effective alternative to conventional vaccines [[105], [106], [107]]. When compared to conventional vaccines, biomimetic nanovaccines have shown advantages in inducing more efficient antigen presentation and immune cell activation, leading to a stronger and more specific immune responses [88,108]. Many comparative studies have revealed that biomimetic nanovaccines can achieve similar or superior levels of immune activation with lower doses of antigens and adjuvants, potentially reducing the risk of adverse side effects [109,110]. Furthermore, the controlled release of antigens and adjuvants from the nanovaccines can provide a more sustained immune response, which is crucial for long-term protection [91,102].

Preclinical studies reveal substantial differences in tumor control capabilities. Biomimetic IL-15 nanovaccines demonstrated superior tumor suppression across multiple models, with 3 out of 6 mice showing complete absence of visible tumors in post-surgical recurrence models [91]. This represents a dramatic improvement over conventional IL-15 therapy, which failed to suppress tumor recurrence adequately. The senescent cancer cell membrane-based nanovaccine (SCCM@NA) showed profound immunological antitumor effects with survival times exceeding 60 days in prophylactic B16-OVA models, compared to significantly shorter survival with conventional senescent cancer cell-based nanovaccines. SCCM@NA achieved enhanced tumor growth delay and longest survival time when compared to bulk mixtures of components [111]. These nanovaccines demonstrated unique advantages in inducing CD8^+^ T cell responses that were completely absent with conventional protein formulations. DLnano-vaccines encoding melanoma Gp100 and Trp2 epitopes induced more potent and consistent epitope-specific CTL responses than DNA monomeric vaccines or CpG-adjuvanted peptide vaccines [112].

The findings from these studies are instrumental in advancing the development of biomimetic nanovaccines for clinical utility, offering new hope for improved cancer treatments and potentially other therapeutic applications. As the field of biomimetic nanovaccines continues to evolve, further research is needed to refine their design and optimize their efficacy. Continued preclinical studies and further clinical trials will be essential in validating the safety and effectiveness of these novel nanovaccines, ultimately leading to their integration into the clinical practice.

### 4.4 Overcoming cancer therapy resistance with biomimetic nanovaccines

Cancer cells evade treatment through multiple interconnected mechanisms [[113], [114], [115], [116]]. Overexpression of ATP-dependent efflux pumps such as P-glycoprotein actively exports chemotherapeutic agents, reducing intracellular drug concentration and leading to multidrug resistance [117]. Enhanced DNA repair pathways allow tumor cells to fix therapy-induced DNA damage, undermining the cytotoxic effects of chemotherapy and radiotherapy [118,119]. Dysregulation of apoptosis via upregulation of Bcl-2 family proteins and activation of survival signaling pathways such as PI3K/AKT prevents programmed cell death, while an immunosuppressive TME, characterized by PD-L1 expression, regulatory T cell recruitment, and cytokines like IL-10, facilitates immune evasion [120]. Cancer stem cells further contribute to resistance by exhibiting intrinsic resistance to apoptosis, elevated DNA repair, and the ability to repopulate tumors after treatment [121,122].

Biomimetic nanovaccines leverage natural cell membranes to enhance antigen presentation and immune activation. These nanoscale platforms typically consist of synthetic cores coated with cancer cell or DCs membranes, preserving native antigen repertoires and surface ligands to evade immune clearance and improve biocompatibility [123]. By bypassing conventional uptake pathways, biomimetic nanovaccines overcome drug efflux systems, as intact nanoparticles are not substrates for P-glycoprotein and other transporters. Incorporation of immunomodulators into the nanovaccine formulation remodels the immunosuppressive microenvironment by repolarizing TAM toward an M1 phenotype and reversing T cell suppression. Furthermore, the presentation of diverse antigens, including cancer stem cell specific markers, enables targeted elimination of therapy-resistant CSC populations, reducing the likelihood of tumor relapse.

Recent advances have introduced pH- and ROS-responsive biomimetic systems that trigger localized antigen and adjuvant release within acidic or oxidative tumor niches. Hybrid membrane technologies, for example combining cancer cell and DCs membranes or integrating exosome-derived vesicles, further enhance targeting and immunogenicity. Early clinical studies of personalized biomimetic nanovaccines, such as antigen-enriched cell membrane@PC7A platforms, demonstrate potent CD8^+^ T cell responses and tumor regression in preclinical models, underscoring their translational potential for overcoming therapy resistance [124].

## 5 Clinical translation and challenges

Following the promising preclinical outcomes of biomimetic nanovaccines, their transition to clinical settings is a critical step toward practical application. This section reviews ongoing clinical trials, translational case studies, and the challenges hindering their widespread adoption, emphasizing the need for robust strategies to bridge the gap between research and patient care.

### 5.1 Current clinical trials

In the realm of biomimetic nanovaccines, many clinical trials are underway, which are meticulously designed to assess immune response, safety profiles, and the overall therapeutic potential of these nanovaccines. In 2021, age-standardized disability-adjusted life-years rates for bladder, kidney, and prostate cancers increased with age [125]. The current clinical evaluation of nanovaccines that mimic the immune system primarily included DCs and tumor cell-based whole-cell nanovaccines [126]. A DC-based cancer vaccine Sipuleucel-T is the first and, as of now, only FDA-approved therapeutic cancer vaccine in 2010 that treat prostate cancer [127,128]. Nevertheless, despite the proven high safety profile of Sipuleucel-T, its clinical efficacy for cancer patients remains uncertain [128]. Safer and effective nanovaccines are being explored in preclinical as well as clinical studies, and the translational investigation of cell membrane-derived nanovaccines is at the forefront of nanomedicine.

Moreover, although tumor cell lysate-associated nanovaccines have shown great promise in clinical trials for modifying tumor cells to express more immune-related factors to produce stronger antitumor immune responses, some other nanovaccines have failed in phase II or III trials due to their insufficient therapeutic effect [126,129]. Gritstone Bio's personalized cancer vaccine failed to produce expected ctDNA changes in a phase 2 trial for metastatic colorectal cancer (NCT05141721) [130]. The vaccine group showed a lower molecular response (30 %) than chemotherapy alone (41.7 %). The company cited a misjudgment in ctDNA dynamics, highlighting the challenge of designing effective trial endpoints. Ultimovacs' cancer vaccine UV1 has failed in three consecutive mid-phase trials across different cancer types, including in mesothelioma, melanoma, and ovarian cancer. Designed to trigger a T-cell response against a common cancer-associated enzyme, UV1 did not improve progression-free survival in head and neck cancer when combined with Keytruda. A key reason may be the reliance on murine models that don't accurately reflect human tumor heterogeneity and immune suppression, leading to overly optimistic trial designs [131]. Obstacles faced by nanovaccine treatment include immune system clearance, insufficient intensity of tumor-specific immune reactions, insufficient targeting of nanovaccine delivery, insufficient stability of formulations, and difficulties in large-scale production.

Furthermore, DCs-derived exosomes, specifically Dex, have also been validated in the clinical settings, demonstrating safety in phase I or II trials for patients with advanced colorectal cancer, melanoma, and non-small cell lung cancer [126,132]. Berger et al. investigated L-asparaginase encapsulated within erythrocytes for elderly patients with Philadelphia chromosome-negative acute lymphoblastic leukemia in a phase II trial, which demonstrated a complete remission rate of 70 %, a median overall survival of 15.8 months, and acceptable toxicity [133]. There remains numerous cell-derived cancer nanovaccines currently being tested in phase I to III clinical trials with promising potential for clinical translational utility, as outlined in Table 1.

**Table 1:** The clinical trials of Nanovaccines in Cancer Therapy.

Title	Cancer types	ID	Year	Status	No. patient	Stage
**GENOCARE: A Prospective, Randomized Clinical Trial of Genotype-Guided Dosing Versus Usual Care**	Colorectal Cancer|Pancreatic Cancer	NCT05391126	9/28/2022	RECRUITING	178	NA
**Rectal Dexmedetomidine Niosomes for Postoperative Analgesia in Pediatric Cancer Patients.**	Postoperative Pain	NCT05340725	5/1/2022	RECRUITING	45	PHASE2|PHASE3
**Nano-SMART: Nanoparticles With MR Guided SBRT in Centrally Located Lung Tumors and Pancreatic Cancer**	Non-small Cell Lung Cancer|Advanced Pancreatic Adenocarcinoma|Unresectable Pancreatic Cancer|Ductal Adenocarcinoma of the Pancreas	NCT04789486	5/27/2021	RECRUITING	100	PHASE1|PHASE2
**BCMA Nano Antibody CAR-T Cells for Patients With Refractory and Relapsed pMultiple Myeloma**	Relapsed and Refractory Multiple Myeloma	NCT03661554 [164]	4/10/2018	COMPLETED	34	EARLY_PHASE1
**Neoadjuvant Pembrolizumab(Pbr)/Nab-Paclitaxel Followed by Pbr/Epirubicin/Cyclophosphamide in TNBC**	Malignant Neoplasm of Breast	NCT03289819 [165]	3/23/2018	COMPLETED	50	PHASE2
**Therapeutic Effect Of Luteolin Natural Extract Versus Its Nanoparticles On Tongue Squamous Cell Carcinoma Cell Line**	Tongue Neoplasms|Carcinoma	NCT03288298	11/1/2017	UNKNOWN	4	EARLY_PHASE1

Apart from ongoing clinical trials, case studies further provide vital real-world insights into the impact of biomimetic nanovaccines on individual patients. In an early case report, Shao et al. observed an overall survival of 10.5 months in a patient with metastatic pancreatic cancer treated with neoantigen nanovaccine and anti-PD-1 antibody [134]. It illustrated that even aggressive, refractory tumors might benefit from personalized nanovaccine approaches combined with immune checkpoint blockade. Robust peptide-specific T-cell responses and sustained functional neoantigen-specific T cell responses were further detected by IFN-γ ELISPOT and intracellular cytokine staining, highlighting the potential for tumor control [134]. Similarly, Sha et al. [135] reported a patient with lung metastases of a phyllodes tumor who achieved a pathological complete response after personalized multi-epitope peptide neoantigen nanovaccine therapy, with the strongest post-vaccine T-cell response detected against the SLC44A5 V54F peptide restricted to HLA-DRB1∗0901. It demonstrated the efficacy of tailored nanovaccines in eliciting potent immune responses against rare tumors. These early-stage clinical case studies on biomimetic nanovaccines for cancer patients could offer a more detailed look at treatment regimens, patient outcomes, and the practical challenges encountered during clinical application.

### 5.2 Challenges and countermeasures in clinical translation

Despite their promise, the clinical translation of biomimetic nanovaccines faces several challenges. These include issues related to manufacturing scalability, ensuring consistent vaccine quality, and addressing regulatory hurdles [123]. Given the high cost of manufacturing and uncontrollable stability, achieving large-scale production of biomimetic nanovaccines with high quality and acceptable batch-to-batch differences poses a significant challenge. For instance, to prevent potential immune responses against endogenous antigens, cellular components used in nanovaccine preparation must be free of heat and pathogen contamination, and sometimes denatured proteins should be eliminated [126]. The manufacturing of nanovaccines should adhere to mature preparation methods, reliable standards, and stringent quality control measures to ensure safety and efficacy. Emerging technologies like flash nanocomplexation offer solutions by enabling rapid, scalable production with homogeneous coatings and narrow polydispersity [136,137]. Flash nanocomplexation achieves high-throughput output and superior colloidal stability compared to traditional methods like sonication, while accommodating diverse core materials and membrane types. However, downstream purification, energy-intensive processes, and the need for standardized protocols for membrane harvesting and antigen loading remain hurdles [138]. Future advancements will require closed-system biomanufacturing with real-time quality monitoring and AI-driven optimization to ensure reproducibility, reduce costs, and meet regulatory standards.

Currently, the International Society of Extracellular Vesicles has developed regulatory considerations for clinical application of therapeutics that are based on extracellular vesicles [123], and regulatory bodies worldwide have established guidelines to oversee the development and approval of these nanovaccines. Meanwhile, substantial input from official faculties, such as the FDA, will be necessary to determine the optimal managerial principles for market approval [123]. Navigating these guidelines while maintaining innovation is a significant challenge for researchers and pharmaceutical companies. Furthermore, complex immune responses elicited by biomimetic nanovaccines are a critical area of study and require more sophisticated monitoring and investigations to fully harness their potential. Understanding how these nanovaccines interact with the immune system at the molecular level, which includes the activation of APCs, T-cell responses, and the generation of memory immune cells, is essential for optimizing their design and function [139].

Overall, biomimetic nanovaccines hold great potential for revolutionizing cancer treatment. While clinical trials and translational case studies are providing valuable insights, the path to widespread clinical application remains complex and challenging, thus requiring continuous research, multidisciplinary approaches, and more mature regulatory measures [140]. Collaboration between researchers, clinicians, manufacturers, and regulatory bodies is crucial to overcome the challenges and unlock the full potential of these innovative nanovaccines [123]. From our perspectives, the integration of advanced technologies such as artificial intelligence and machine learning algorithm may further enhance the development and clinical translation of biomimetic nanovaccines in the future. Personalized medicine and precision cancer treatments could become more accessible, offering tailored regimens to individual patients based on their unique genetic and immunological profiles.

## 6 Perspectives and conclusion

As biomimetic nanovaccines navigate the complexities of clinical translation, their future potential and societal implications warrant careful consideration. This section offers perspectives on advancing their development, addressing accessibility and cost challenges, and concludes with key insights to guide their integration into precision oncology. Biomimetic nanovaccines represent a paradigm shift in the field of cancer immunotherapy, leveraging the principles of nanotechnology and biomimicry to enhance immune responses against tumors. These innovative nanovaccines, which mimic the natural structures and functions of biological systems, have demonstrated significant potential in preclinical models, offering new avenues for the development of more effective and personalized cancer therapies.

One of the critical advantages of biomimetic nanovaccines is their ability to precisely target tumor cells while simultaneously activating the immune system. By incorporating elements such as cell membranes, viral-like particles, or other biomimetic materials, these nanovaccines can achieve enhanced delivery and presentation of tumor antigens, leading to a more robust and sustained immune response. This targeted approach not only improves the efficacy of the nanovaccines but also reduces off-target effects and minimizes toxicity, which are significant limitations of traditional cancer treatments.

Biomimetic nanovaccines show promise as prophylactic agents due to their ability to generate long-lasting immunogenic memory against cancer-associated antigens at lower doses with reduced adjuvant requirements, thereby mitigating associated toxicities [141]. The technology's capacity to deliver nanoparticles that enhance the immune system's ability to recognize and eliminate tumors makes them ideal candidates for preventing cancer development in high-risk populations.

Recent single-cell and spatial transcriptomic analyses in oncology highlight tumor heterogeneity and personalized immune activation as key variables, factors that biomimetic nanovaccine platforms can be engineered to address [142]. The nanovaccine platforms can be rapidly customized based on individual tumor profiles identified through single-cell analysis. The personalized neoantigen nanovaccine platform demonstrates this capability, showing superior protective efficacy against tumor recurrence when combined with anti-PD-1 treatment [143]. Biomimetic nanovaccines can simultaneously present multiple tumor antigens, addressing the challenge of tumor heterogeneity by targeting diverse cancer cell populations [144]. In addition, advanced nanovaccine designs incorporate spatiotemporal control of immune activation, allowing for precise delivery of antigens and adjuvants to specific tissue locations [19]. The future of cancer immunotherapy lies in this integrated approach, where molecular insights drive therapeutic design, and spatial information guides delivery strategies.

Artificial intelligence has fundamentally transformed vaccine design through the development of sophisticated computational frameworks. The breakthrough LinearDesign AI tool, published in Nature in 2023 and refined through 2024–2025, represents a paradigm shift in mRNA vaccine optimization [145]. This AI-driven approach has resulted in vaccines that generate antibody responses up to 128 times greater than traditional methods, attributed to increased mRNA sequence stability that maintains integrity longer due to its folded, DNA-like structure. The field has witnessed unprecedented innovation in adjuvant combinations that harness multiple immune pathways simultaneously. Recent developments include the creation of STING agonist-based ER-targeting molecules that deliver antigens directly to the ER of DCs, substantially enhancing CD8^+^ T cell immune responses [146]. The Alhydroxiquim-II adjuvant system, the first TLR7/8 agonist approved in an infectious disease vaccine, demonstrates exceptional safety profiles by targeting potent immunostimulators directly to draining lymph nodes [147]. The emergence of personalized nanovaccine platforms represents a paradigm shift toward precision medicine approaches. Antigen-enriched tumor cell membrane nanovaccines coupled with PC7A adjuvant demonstrate the ability to induce robust poly-neoepitopic T-cell responses even at low dosages, achieving significant tumor regression and metastasis inhibition across multiple cancer models [124]. The convergence of these biomimetic technologies positions nanovaccines at the forefront of next-generation immunization strategies. Current clinical trials demonstrate the translational potential of exosome-based platforms, STING-activating adjuvants, and AI-designed delivery systems [148,149].

Another area that requires further exploration is the interaction between biomimetic nanovaccines and the TME. The TME plays a crucial role in modulating immune responses, often creating an immunosuppressive environment that hinders the effectiveness of cancer therapies [150]. Future research should focus on understanding how biomimetic nanovaccines can be engineered to overcome these immunosuppressive barriers, potentially through combination therapies that target multiple aspects of the TME. Looking ahead, the integration of biomimetic nanovaccines with other therapeutic modalities, such as immune checkpoint inhibitors, radiotherapy, or even emerging technologies like CRISPR-based gene editing, could pave the way for more comprehensive and effective cancer treatment strategies. These combination strategies address the immunosuppressive mechanisms employed by cancer cells that often limit the effectiveness of single-agent therapies [151]. Biomimetic nanovaccines can transform “cold” TME into “hot” immune-infiltrated environments, making them more susceptible to other immunotherapeutic interventions [95,144]. Studies demonstrated that co-administration of nanovaccines with checkpoint inhibitors significantly increases treatment efficacy compared to checkpoint inhibitor monotherapy. In melanoma models, combination therapy achieved 87.5 % response rates with complete remissions, compared to limited efficacy of checkpoint inhibitors alone [152]. Chronobiological factors, such as circadian regulators, significantly modulate immune cell metabolism and tumor microenvironments, insights that could optimize vaccine timing or enhance efficacy when paired with nanovaccines [153].

Nanotechnology presents versatile opportunities to modulate the microbiome and enhance cancer treatment efficacy [154]. The interplay between the microbiome and tumor immunity reveals potential for combining microbiome-targeted strategies with vaccine adjuvants to boost anti-tumor responses [155,156]. For example, probiotic formulations paired with cancer nanovaccines have shown synergistic effects in activating both innate and adaptive immunity [157]. Engineered probiotics have been developed for local tumor delivery of checkpoint blockade nanobodies, offering more targeted immunotherapy with reduced systemic toxicity [158]. Additionally, gut microbiota depletion has been shown to dramatically enhance nanoparticle accumulation in tumors through increased vascular permeability [159]. Nanoparticle-mediated targeting of specific oncogenic bacteria represents a dual-approach strategy that simultaneously eliminates cancer-promoting microbes while delivering anti-cancer therapeutics [160].

Recent groundbreaking research has revealed significant age-associated disparities in nanomedicine effectiveness. A pivotal study published in Nature Nanotechnology demonstrated that cancer nanomedicine shows superior tumor delivery and treatment outcomes in older versus younger patients, because of age-related decline in hepatic phagocytic clearance [161]. One of the most promising applications of nanomedicine in aging-related cancer involves targeting senescent cells. These cells, which accumulate with age, contribute to cancer development through the senescence-associated secretory phenotype [162]. Advanced nanoparticle systems have been developed to specifically target senescent cells using biomarkers like β-galactosidase [163]. The future of nanomedicine in aging populations lies in personalized approaches, for example, age-specific nanoparticle design accounting for physiological changes and precision targeting based on senescence markers and aging pathways.

Additionally, the principles underlying biomimetic nanovaccines could be extended beyond oncology, offering new therapeutic approaches for infectious diseases, autoimmune disorders, and other conditions where immune modulation is key.

In conclusion, while biomimetic nanovaccines hold tremendous promise, their successful translation into clinical practice will require a concerted effort across multiple disciplines, including nanotechnology, immunology, materials science, and clinical research. The potential benefits of these nanovaccines, from improved efficacy to reduced side effects, underscore the importance of continued investment and innovation in this rapidly evolving field. As research progresses, biomimetic nanovaccines are poised to become a cornerstone of precision oncology, offering new hope and more effective treatment options for cancer patients.

## CRediT authorship contribution statement

**Dilinaer Wusiman:** Writing – review & editing, Writing – original draft, Validation, Software, Methodology, Formal analysis, Data curation, Conceptualization. **Yu Wang:** Writing – review & editing, Writing – original draft, Validation, Software, Methodology, Investigation, Formal analysis, Conceptualization. **Minghao Wang:** Writing – review & editing, Writing – original draft, Visualization, Validation, Software, Resources, Investigation, Formal analysis, Data curation. **Jie Wang:** Writing – review & editing, Validation, Software, Resources, Investigation, Data curation. **Ruicheng Wu:** Writing – review & editing, Validation, Software, Resources, Methodology, Data curation. **Zhouting Tuo:** Writing – review & editing, Validation, Software, Resources, Methodology, Data curation. **Zhipeng Wang:** Writing – review & editing, Validation, Methodology, Investigation, Data curation. **Qingxin Yu:** Writing – review & editing, Software, Resources, Methodology, Data curation. **Zhaohong An:** Writing – review & editing, Software, Resources, Methodology, Formal analysis, Data curation. **William C. Cho:** Writing – review & editing, Validation, Project administration, Methodology, Formal analysis, Data curation, Conceptualization. **Dengxiong Li:** Writing – review & editing, Validation, Supervision, Project administration, Methodology, Investigation, Data curation, Conceptualization. **Wuran Wei:** Writing – review & editing, Supervision, Resources, Project administration, Data curation, Conceptualization. **Dechao Feng:** Writing – review & editing, Validation, Supervision, Project administration, Investigation, Formal analysis, Data curation, Conceptualization.

## Declarations

The authors utilized AI-assisted editing tool (Grammarly, ChatGPT-4) solely for language refinement purposes, including grammar checking, sentence structure optimization, and vocabulary enhancement. All conceptual development, technical content, and critical analysis remain entirely human-generated. The authors take full responsibility for the accuracy of information and academic integrity of this work.

## Ethics approval and consent to participate

Not available.

## Consent for publication

Not available.

## Funding declaration

No funding.

## Declaration of competing interest

The authors declare that they have no known competing financial interests or personal relationships that could have appeared to influence the work reported in this paper.

## Data Availability

No data was used for the research described in the article.
